# Effectiveness of Social Problem-Solving Interventions for Children with Autism Spectrum Disorder: A Systematic Review and Meta-Analysis

**DOI:** 10.3390/bs15121708

**Published:** 2025-12-10

**Authors:** Shaoju Jin, Sheng Xu, Yu Zhao, Huan Huang, Han Zhu, Chunyan Zhou

**Affiliations:** 1 Faculty of Education, East China Normal University, Shanghai 200062, China; 52274109003@stu.ecnu.edu.cn (S.J.); 51274109001@stu.ecnu.edu.cn (Y.Z.); 52284109002@stu.ecnu.edu.cn (H.H.); zhuhan_007@stu.ecnu.edu.cn (H.Z.); 2 Sichuan Revolutionary Old Area Development Research Center, Sichuan University of Arts and Science, Dazhou 635000, China; 20180040.z-cy_@sasu.edu.cn

**Keywords:** autism spectrum disorder, social problem-solving, social-emotional learning, meta-analysis, teacher-led intervention, school-based implementation, ecological validity

## Abstract

Social problem-solving (SPS) is a core component of social-emotional learning (SEL) that integrates cognitive, emotional, and behavioral processes essential for adaptive social functioning. Children with autism spectrum disorder (ASD) often experience persistent difficulties in these domains, highlighting the need for effective interventions. This meta-analysis quantitatively synthesized evidence on the effectiveness of SPS interventions for children with ASD. Nineteen group-design studies involving 741 participants met inclusion criteria. Using random-effects models, the pooled results revealed a significant, moderate overall effect on SPS competence (Cohen’s *d* = 0.53, 95% CI [0.15, 1.01], *p* < 0.05). Subgroup analyses further indicated that teacher-led and school-based implementations produced stronger effects than researcher-led interventions in non-school contexts, underscoring the importance of ecological validity. SPS interventions also generated moderate-to-large improvements in related SEL domains, including social skills, emotion recognition, theory of mind, and executive function. These findings support SPS as a pivotal mechanism for promoting social-emotional development in children with ASD. Future research should employ more rigorous designs, report implementation fidelity, and examine the sustainability of teacher-led interventions within naturalistic school settings.

## 1. Introduction

Social and emotional skills acquired in childhood are critical predictors of lifelong well-being. During this key stage, children develop the ability to regulate emotions, understand others’ perspectives, and resolve social problems. These abilities provide a foundation for positive peer relationships, academic success, and mental health (Domitrovich et al., 2017; Jones et al., 2017). However, children and adolescents with autism spectrum disorder (ASD) often struggle in these areas. They face challenges with social-emotional reciprocity, nonverbal communication, and building relationships (American Psychiatric Association, 2013). In school settings, these difficulties can lead to peer rejection and isolation, making it hard to form friendships (Bauminger & Kasari, 2000; Bauminger et al., 2003). Without proper support, these social challenges can worsen, increasing the risk for social withdrawal, behavior problems, and related mental health conditions (Tantam, 2003; Locke et al., 2017; Reid et al., 2020).

The framework of Social and Emotional Learning (SEL) offers a systematic approach to cultivating these essential competencies. According to the Collaborative for Academic, Social, and Emotional Learning (CASEL), SEL comprises five core domains: self-awareness, social awareness, self-management, responsible decision-making, and relationship skills (CASEL, 2018). Within this framework, Social Problem-Solving (SPS) emerges as a central integrative skill. Unlike broad social skills training that often focuses on teaching discrete behaviors, SPS is distinct in its focus on the underlying cognitive processes (McNair et al., 2024). SPS refers to the cognitive behavioral process individuals use to identify, evaluate, and resolve interpersonal challenges (Nezu, 2004). Conceptually, SPS acts as a central integrative skill that links cognition (e.g., executive control), emotion (e.g., regulation), and behavior. It links core cognitive processes such as flexibility, perspective-taking, and executive control, which are frequently areas of difficulty for children with ASD (Baron-Cohen et al., 1985; Mazefsky & White, 2014). Accordingly, explicit instruction in SPS offers a structured pathway for these children to develop adaptive reasoning (McNair et al., 2024). SPS instruction typically progresses through a cognitive-emotional-behavioral sequence that includes identifying the social problem and contextual cues, interpreting others’ intentions and emotions, generating alternative solutions, evaluating the potential consequences of each option, and selecting and enacting an adaptive response. This sequence illustrates how SPS skills intersect with multiple social and cognitive domains and why SPS-based interventions may support broad improvements in social–emotional functioning.

SPS interventions are typically grounded in established theoretical frameworks. The Social Information Processing Model conceptualizes social decision-making as a stepwise process of cue encoding, interpretation, response generation, and evaluation (Crick & Dodge, 1994). The Problem-Solving Therapy Model emphasizes the influence of cognitive orientation and strategy use on adaptive problem resolution (Nezu, 2004). The Interpersonal Cognitive Problem-Solving Model applies structured training to teach children to generate and evaluate multiple social solutions (Spivack & Shure, 1976). These frameworks conceptualize SPS as a multistep cognitive-behavioral process. Accordingly, most SPS interventions use strategies such as direct instruction, modeling, and role-playing to teach these steps. Although initial studies have shown promising effects (Bauminger, 2002; Bonete et al., 2015, 2016, 2022; Szumski et al., 2019), the broader evidence base has yet to be systematically synthesized.

Although SEL research within ASD education has grown rapidly, existing systematic reviews and meta-analyses primarily address adjacent domains. For instance, reviews of social skills interventions (Alahmari et al., 2025; Bellini et al., 2007; Gates et al., 2017; Miller et al., 2014) typically focus on broad skill-building programs that do not specifically target the multistep reasoning processes involved in SPS. Reviews of emotion recognition (Berggren et al., 2018; Zhi et al., 2021), self-management (Carr et al., 2014; Scheibel et al., 2024), and theory of mind (Fletcher-Watson et al., 2014) examine related constructs but do not isolate SPS as a core instructional focus. While these reviews have established the efficacy of broad social interventions, they often treat social skills training as a monolithic category, overlooking the specific cognitive mechanisms. Similarly, reviews on isolated skills like emotion recognition or theory of mind do not address how these component skills are integrated into the complex decision-making required for real-world social adaptation.

To date, no systematic review or meta-analysis has specifically synthesized the evidence for interventions that feature SPS as a core component, although related meta-analyses have examined overlapping constructs. This gap is particularly notable given that recent evidence has revealed that discrete SPS components, such as problem identification and solution evaluation, contribute uniquely and jointly to social functioning in autistic youth (McNair et al., 2024). These findings underscore the need for comprehensive analyses that capture both the overall efficacy of SPS interventions and the mechanisms underlying their effects. Consequently, there is an urgent need for a comprehensive quantitative synthesis to evaluate the efficacy of these interventions and pinpoint key moderators influencing their outcomes.

To address these gaps, the present study conducted the first systematic review and meta-analysis of SPS-based interventions for children and adolescents with ASD. By synthesizing quantitative evidence across multiple domains, this study aims to evaluate the overall efficacy of SPS interventions, examine contextual and implementation moderators, and assess the methodological quality of existing research. The research questions of this study include the following:

(1) What are the common characteristics of SPS-based interventions for youth with ASD (e.g., participants, components, and settings)?

(2) What are their overall effects on SPS competence and related SEL abilities?

(3) Which contextual and implementation factors (e.g., setting, implementer type, intervention duration) moderate intervention outcomes?

## 2. Materials and Methods

This systematic review and meta-analysis followed the Preferred Reporting Items for Systematic Reviews and Meta-Analyses (PRISMA) 2021 guidelines (Table S6) (Page et al., 2021) and was pre-registered with the International Prospective Register of Systematic Reviews (PROSPERO, CRD420251165241).

### 2.1. Search Strategy

A systematic literature search was conducted on 17 March 2025, across seven core electronic databases to identify relevant studies published up to that date. Databases included ERIC (ProQuest), EBSCOhost (encompassing PsycINFO, Academic Search Premier, Psychological and Behavioral Sciences Collection, and Education Full Text), Social Science Citation Index (Web of Science), and PsycNET (PsycARTICLES). The search focused on title, abstract, and subject fields, using Boolean operators and truncation (asterisk *) to capture lexical variants and different word forms. The search strategy combined three conceptual blocks: (1) Population terms: “autism*” OR “autistic” OR “ASD” OR “HFA” OR “Asperger” OR “PDD”; (2) Intervention targets: “social problem solving” OR “interpersonal problem solving” OR “problem-solving skills” OR “cognitive-behavior*” OR “social emotional learning” OR “conflict problem resolution”; and (3) Intervention type: “intervention*” OR “training” OR “program” OR “therapy” OR “treatment”.

Boolean operators AND/OR were used to combine these blocks to ensure comprehensive retrieval of relevant studies. Search strings were adapted to the specific syntax and indexing rules of each database. A full record of database-specific search strings, syntax specifications, and the number of records retrieved from each database is provided in Supplementary Materials (Table S1). In addition, reference lists of identified articles, previous systematic reviews, and meta-analyses were manually screened to identify further eligible studies. No language restrictions were applied, although only studies with full-text availability in English were included in the quantitative synthesis. Although we aimed for comprehensive coverage, we prioritized peer-reviewed journals to ensure the methodological quality of the evidence. Grey literature, such as dissertations and conference abstracts, was screened but ultimately excluded if it had not been published in a peer-reviewed venue. This rigorous search process ensured comprehensive coverage of both published and peer-reviewed research relevant to SPS interventions for children and adolescents with ASD.

### 2.2. Inclusion and Exclusion Criteria

Studies were included if they met the following criteria. (1) They were published in peer-reviewed journals between January 1990 and January 2025. Non-peer-reviewed articles, conference presentations, dissertations, and studies not written in English were excluded to ensure the reliability and accessibility of data. (2) Participants were children and adolescents aged 4–18 years with a formal diagnosis of ASD. Studies that primarily included participants with attention-deficit/hyperactivity disorder, other developmental disorders, or typically developing children were excluded, in order to maintain focus on the target population. (3) Studies employed a group-based experimental design, including randomized controlled trials (RCTs) and quasi-experimental studies with a comparison or control group. Single-case designs, descriptive studies, qualitative research, and literature reviews were excluded to ensure methodological rigor and comparability across studies. (4) The independent variable was defined as any intervention incorporating SPS components grounded in behavioral, cognitive-behavioral, or social-cognitive frameworks. Interventions that focused solely on inclusion policies or environmental modifications without direct SPS instruction were excluded. (5) The dependent variables included SPS ability and related social-emotional competencies, such as social skills, emotion recognition, theory of mind, and executive function. Studies were required to report quantitative results for at least one of these domains, reflecting measurable improvements in SEL among children with ASD. (6) Studies needed to provide sufficient statistical information to calculate standardized effect sizes (Cohen’s *d*). Eligible studies reported either group means and standard deviations at post-test, or sample sizes and inferential statistics (e.g., *t*, *F*, or *χ*^2^ values) that could be converted into standardized mean differences. Studies lacking adequate data for effect size computation were excluded.

Together, these criteria ensured that the meta-analysis synthesized only rigorous, quantitatively analyzable studies that directly examined SPS-related social-emotional outcomes in children and adolescents with ASD.

### 2.3. Data Extraction and Coding

The coding form was designed to capture both descriptive and quantitative information necessary for meta-analytic synthesis. The coding form included participant demographics, intervention details, study characteristics, outcome measures, and all relevant statistical data required to calculate standardized effect sizes. The full descriptions of all coded variables, operational definitions, and coding rules are presented in Supplementary Materials (Table S2). Data extraction was conducted by five trained coders, three doctoral students and two co-authors, who followed a structured training protocol. Coding disagreements were resolved through discussion and consensus meetings (Burla et al., 2008). To balance reliability and efficiency, 15% of studies were triple-coded, 28% were double-coded, and the remaining studies were coded independently. Inter-coder reliability was excellent, with Cohen’s kappa reaching κ = 0.89 for triple-coded studies and κ = 0.87 for double-coded studies, indicating substantial to near-perfect reliability consistent with established benchmarks (Landis & Koch, 1977; κ > 0.81 = almost perfect, 0.61–0.80 = substantial).

### 2.4. Study Quality and Risk of Bias Assessment

Methodological quality and risk of bias were assessed using the What Works Clearinghouse (WWC) Standards Handbook, Version 4.1 (What Works Clearinghouse, 2020). The WWC framework evaluates the internal validity of group design studies across five key domains: (1) randomization; (2) participant attrition; (3) baseline equivalence between groups; (4) reliability and validity of outcome measures; (5) confounding factors. Based on these domains, each study was assigned one of three ratings: Meets WWC Standards Without Reservations, Meets WWC Standards with Reservations, or Does Not Meet WWC Standards. This classification provides a systematic and transparent method for assessing the overall methodological rigor and the strength of evidence contributed by each study. By applying the WWC standards, we ensured a consistent and replicable evaluation of study quality, which not only informed interpretation of the meta-analytic results but also helped identify potential sources of heterogeneity across studies. This approach strengthens confidence in the validity of synthesized findings and aligns with best practices for rigorous evidence synthesis in behavioral intervention research.

### 2.5. Data Synthesis and Statistical Analysis

All statistical analyses were conducted using Comprehensive Meta-Analysis (CMA) 3.0. Prior to synthesis, data were screened for outliers through visual inspection of forest plots, with no extreme values identified (Baker & Jackson, 2008). To ensure consistent interpretation across studies, all outcomes were coded so that a positive effect size uniformly indicated an improvement in the target skill. Standardized mean differences (Cohen’s *d*) with 95% confidence intervals (CIs) were calculated for each outcome. Effect sizes were calculated from post-test means and standard deviations when available, or estimated from other reported statistics (e.g., *t*-values, *p*-values) when necessary (Wilson & Lipsey, 2001). Standardized effect sizes were interpreted following conventional guidelines, with values of 0.20, 0.50, and 0.80 representing small, medium, and large effects, respectively (Lakens, 2013).

Heterogeneity was examined using the Cochrane Q and *I*^2^ statistics. Conventionally, *I*^2^ values of 25%, 50%, and 75% are interpreted as indicating low, moderate, and high heterogeneity, respectively (DerSimonian & Laird, 1986). Outcome data were pooled using a random effects model to account for expected variability in study characteristic and interventions. Sensitivity analyses were conducted using fixed effects models to verify the robustness of the findings when heterogeneity was low (Higgins et al., 2003).

Sensitivity analysis and potential publication bias were evaluated using leave-one-out sensitivity analyses, funnel plots, and Egger’s regression test (*p* < 0.05 indicating potential bias) (Egger et al., 1997). The fail-safe N was calculated to estimate the number of missing null studies needed to nullify the observed effects (Rosenthal, 1979). When evidence of bias was detected, the trim-and-fill procedure was applied to generate adjusted pooled estimates (Duval & Tweedie, 2000). This comprehensive analytic approach ensured accurate estimation, accounted for between-study variability, and strengthened confidence in the validity of findings regarding the efficacy of SPS interventions for children and adolescents with ASD.

## 3. Results

### 3.1. Study Selection

**Figure 1:**
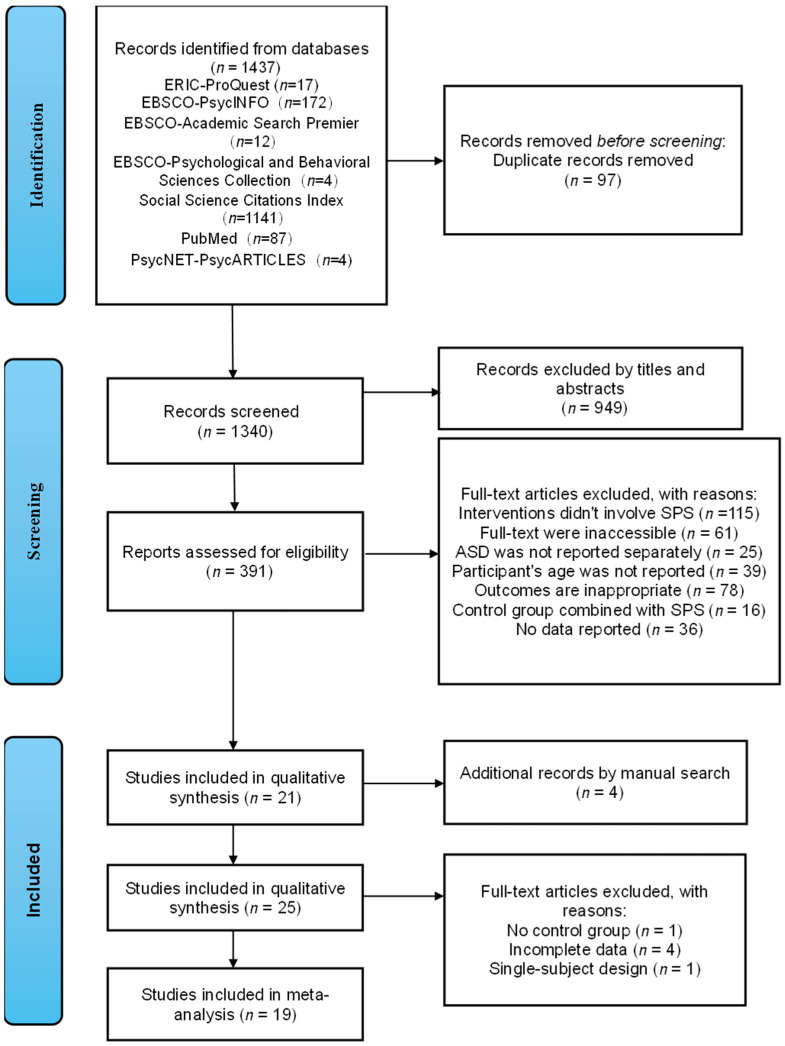
The flow diagram of study selection procedure.

The study selection process is illustrated in the PRISMA flow diagram (Figure 1). The initial database search yielded 1437 records, which were reduced to 1340 after duplicate removal. These records underwent a systematic multi-stage screening process, including title, abstract, and full-text review. In addition, a manual search of reference lists identified four additional eligible articles. Following this comprehensive screening, 25 studies met the inclusion criteria and were retained for qualitative synthesis. Six studies were excluded from the quantitative analysis due to insufficient statistical data or the absence of a control or comparison group, resulting in a final sample of 19 studies included in the meta-analysis.

### 3.2. Characteristics of Included Studies

To address Research Question 1, the characteristics of the included studies were synthesized and summarized (Table 1).

#### 3.2.1. Participant and Study Characteristics

Nineteen intervention studies involving a total of 741 participants with ASD were included. The overall sample was predominantly male (88%). Participants’ ages ranged from 4 to 18 years, with most studies focusing on school-aged children (6–12 years; *n* = 11), followed by adolescents (12–18 years; *n* = 6) and preschoolers (4–6 years; *n* = 2). The majority of studies were conducted in high-income countries, particularly Israel (*n* = 6) and the United States (*n* = 6). In terms of research design, 10 studies employed quasi-experimental methods, whereas nine used RCTs. Reporting of key methodological features varied: 11 studies (58%) reported treatment fidelity, six (32%) included follow-up or maintenance assessments, and eight (42%) assessed social validity. Although the evidence base is growing, inconsistencies in methodological reporting limit comparability across studies.

#### 3.2.2. Intervention Characteristics

Interventions were delivered in various settings, most frequently in schools (*n* = 11). The predominant delivery format was small-group instruction (*n* = 16), typically involving three to five participants per group. Intervention agents varied and included teachers (*n* = 8), researchers (*n* = 5), graduate students (*n* = 5), and collaborative teams (*n* = 8). Intervention dosage demonstrated substantial variability: the most common frequency was one session per week (*n* = 13), sessions typically lasted 20–60 minutes (*n* = 11), and total intervention duration most often ranged from 12 to 28 weeks (*n* = 11). This variation reflects differences in program design and feasibility across educational and clinical contexts.

#### 3.2.3. SPS Components and Implementation Roles

The role of the SPS component was classified into three categories:

(1) Primary Focus (*n* = 6): Programs such as the *I Can Problem Solve* curriculum directly taught foundational concepts, emotion recognition, and interpersonal problem-solving steps to enhance children’s SPS skills (Bauminger, 2002; Szumski et al., 2019). Other cognitive-behavioral therapy-based programs used computer-assisted formats or role-playing to train conflict negotiation and problem-solving (Bonete et al., 2016; Hochhauser et al., 2018; Eden & Oren, 2021).

(2) Component of SEL curriculum (*n* = 9): SPS was embedded within broader SEL frameworks such as Social Skills Training (Bauminger, 2007a, 2007b), Secret Agent Society (Einfeld et al., 2018), and the Social Competence Intervention (Stichter et al., 2010, 2012). These typically integrated SPS within cognitive-behavioral therapy-based SEL curricula to promote generalizable competencies (Koning et al., 2013; Lee et al., 2019).

(3) Component of multi-component interventions (*n* = 4): SPS was one of several coordinated therapeutic components. These comprehensive programs integrated SPS instruction with a focus on related domains, such as targeting executive function deficits (e.g., flexibility, planning) or using engaging formats like game-based curricula and computer-based vignettes to teach a combination of social and problem-solving skills (Beaumont & Sofronoff, 2008; Bauminger et al., 2013; Kenworthy et al., 2014; Chou, 2024).

#### 3.2.4. Outcome Variables and Informants

The most frequently evaluated outcomes were social skills (*n* = 16), SPS competence (*n* = 14), and emotion recognition (*n* = 13). These domains were predominantly assessed through informant reports, with teacher ratings serving as the primary data source. In contrast, theory of mind was assessed in eight studies, most often using direct performance-based measures (*n* = 6). Executive function outcomes appeared in four studies, relying mainly on informant-based evaluations. This heavy reliance on subjective informant measures, particularly for social domains, highlights a potential source of measurement bias and underscores the need for more standardized, performance-based assessments in future research. This distribution highlights a continued reliance on subjective measures, particularly for social domains, and underscores the need for more standardized, performance-based assessments in future research.

### 3.3. Methodological Quality and Risk of Bias

The methodological quality of the 19 included studies was evaluated according to the WWC Standards Handbook (Version 4.1). A detailed breakdown of study ratings is presented in Supplementary Materials (Table S3). Six studies met WWC Standards without reservations, typically RCTs characterized by low attrition and baseline equivalence. Another six studies met WWC Standards with reservations, most often due to methodological concerns such as high participant attrition or unaddressed group differences at baseline. The remaining seven studies did not meet WWC Standards, primarily because they relied on single-group pre–post designs lacking a control or comparison condition. Overall, nearly two-thirds demonstrated at least moderate rigor, though variability underscores the need for stronger adherence to reporting standards.

### 3.4. Overall Effects of SPS Interventions

To address Research Question 2, a meta-analysis examined the effects of SPS interventions on SPS competence and related SEL domains. Individual effect sizes are listed in Supplementary Table S4.

#### 3.4.1. Effect on SPS Competence

**Figure 2:**
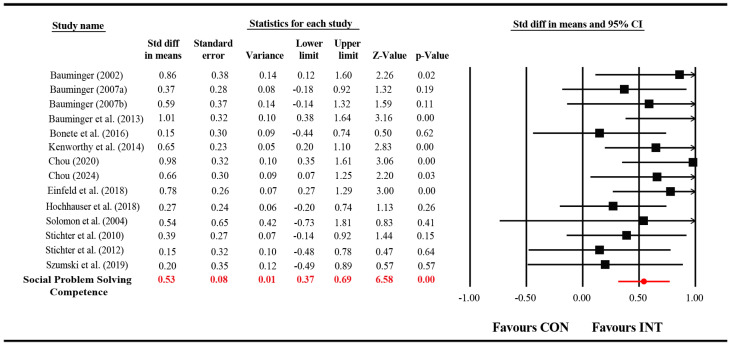
Forest plot of SPS competence outcomes. Note: The statistics displayed in red represent the overall pooled effect size derived from the fixed-effects model.

Fourteen studies (*k* = 14) assessed SPS competence. Heterogeneity was non-significant, *Q* (13) = 12.02, *p* = 0.52, *I*^2^ = 0.0%, supporting a fixed-effects model. The pooled standardized mean difference indicated a moderate positive effect (*d* = 0.53, SE = 0.08, 95% CI [0.15, 1.01], *p* < 0.05) (Figure 2). These findings show that SPS-targeted interventions significantly improved participants’ ability to identify, generate, and apply social solutions. The low heterogeneity (*I*^2^ = 0.0%) suggests a remarkable consistency in the intervention’s effect on this core cognitive skill across diverse study settings and populations, reinforcing the robustness of SPS training.

The modest magnitude likely reflects the complexity of SPS, which requires repeated practice and contextual generalization.

#### 3.4.2. Effects on SEL-Related Competence

**Figure 3:**
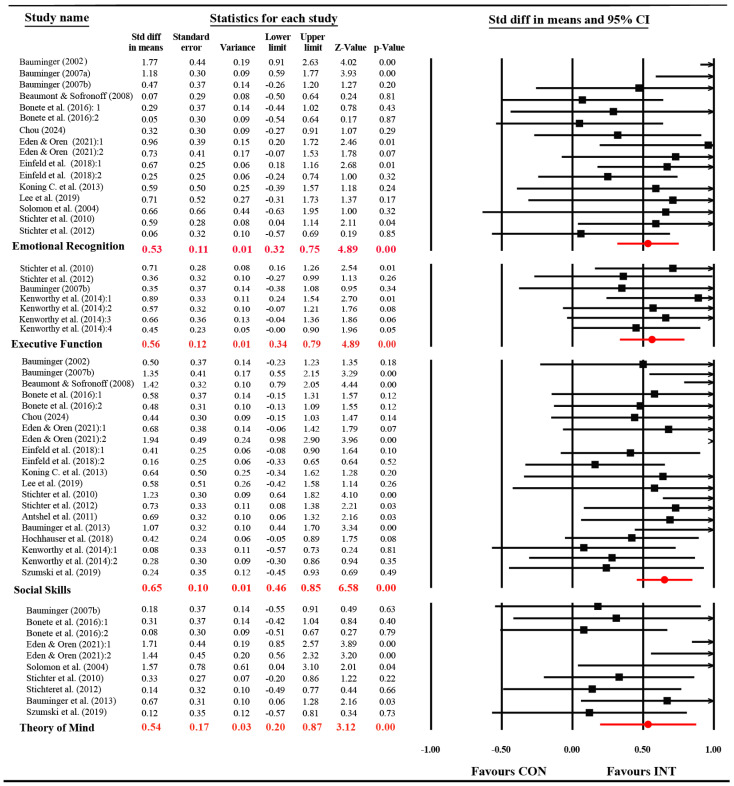
Summary of pooled effects across SEL-related domains. Note: The statistics displayed in red represent the overall pooled effect size estimates for each outcome domain. Citations followed by numeric suffixes (e.g., :1, :2) indicate distinct effect sizes extracted from a single study.

To further address Research Question 2, separate meta-analyses were conducted across four SEL-related outcome domains: emotion recognition, executive function, social skills, and theory of mind. The pooled results are summarized in Figure 3.

(1) Emotional Recognition: Thirteen studies (k = 16) reported emotion recognition outcomes, showing moderate heterogeneity (Q (15) = 24.00, *p* = 0.06; *I*^2^ = 37.50%). The meta-analysis revealed a significant medium effect (*d* = 0.53, SE = 0.10, 95% CI [0.32, 0.74], *p* < 0.05). This result indicates that SPS-based activities, which often involve interpreting social cues, may successfully facilitate broader emotion decoding skills.

(2) Executive Function: Four studies (k = 7) assessed executive function outcomes and showed no heterogeneity (*I*^2^ = 0.0%). A fixed-effect model indicated a medium-sized significant effect (*d* = 0.56, 95% CI [0.35, 0.89], *p* < 0.001). Sensitivity analysis using a random-effects model yielded nearly identical results, confirming the robustness of this effect. Similarly to SPS competence, the absence of heterogeneity (*I*^2^ = 0.0%) implies that the impact of SPS interventions on executive function is highly consistent across studies, supporting the theoretical link between problem-solving and executive control.

(3) Social Skills: Sixteen studies (k = 20) examined social skills outcomes. The heterogeneity test indicated moderate variability (Q (19) = 33.60, *p* = 0.02; *I*^2^ = 43.61%), supporting the use of a random-effects model. The pooled estimate revealed a significant moderate-to-large effect (*d* = 0.65, SE = 0.10, 95% CI [0.24, 1.42], *p* < 0.05). This finding underscores the practical relevance of SPS-based instruction for real-world social functioning, although the moderate heterogeneity suggests that effectiveness may vary depending on contextual factors.

(4) Theory of Mind: Eight studies (k = 10) evaluated theory of mind outcomes, with moderate heterogeneity (Q (9) = 19.81, *p* = 0.01; *I*^2^ = 54.57%). The random-effects analysis revealed a significant, medium effect (*d* = 0.54, SE = 0.17, 95% CI [0.08, 1.71], *p* < 0.01). The pattern aligns with the conceptual overlap between theory of mind and SPS. Both require perspective taking and inferential reasoning about others’ intentions. These results highlight that SPS-focused interventions may indirectly enhance theory of mind through repeated practice in social reasoning and reflective dialogue.

### 3.5. Moderator Analyses of SPS Effects

To address Research Question 3, subgroup analyses were conducted to identify moderators influencing the effectiveness of SPS interventions (see Table 2). A significant moderation effect emerged for implementer type (Q_B_ (2) = 7.31, *p* = 0.03), with teacher-led (*d* = 0.72) and collaborative (*d* = 0.63) programs yielding stronger effects than researcher-led interventions (*d* = 0.20). This finding highlights the critical role of practitioners, whose ongoing engagement and ecological familiarity with the classroom environment may facilitate the generalization and maintenance of skills better than transient researcher-led sessions. A marginally significant moderation was also found for setting (Q_B_ (1) = 3.46, *p* = 0.06), as school-based programs (*d* = 0.61) outperformed those in non-school contexts (*d* = 0.26), further suggesting that ecological validity and opportunities for real-world practice are key drivers of success. No other moderators including dosage variables (frequency, session length, total duration) or SPS component type reached statistical significance, though longer and more integrated interventions tended to show larger effects.

### 3.6. Publication Bias and Sensitivity Analyses

**Figure 4:**
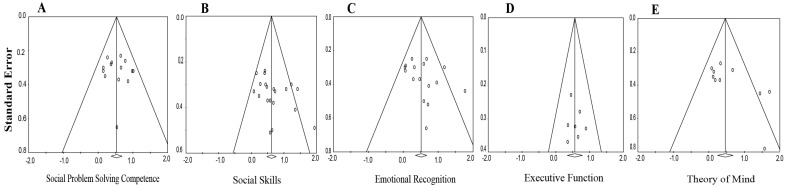
Funnel plots for publication bias across outcome domains. *Note*. (**A**) Social Problem-Solving Competence; (**B**) Social Skills; (**C**) Emotion Recognition; (**D**) Executive Function; and (**E**) Theory of Mind. Each circle represents the effect size (Cohen’s *d*) from an individual study, plotted against its standard error (y-axis). The vertical solid line indicates the pooled summary effect size, and the diagonal lines represent the pseudo 95% confidence interval.

Publication bias was examined both SPS competence and related SEL domains using funnel plots (Figure 4), Egger’s regression tests, and trim-and-fill corrections (see Supplementary Table S5). Results varied by outcome. No bias was detected for SPS competence (Egger’s test, *p* = 0.74), indicating strong confidence in the primary effect. For SEL-related outcomes, potential asymmetry was observed in social skills (*p* = 0.064) and theory of mind (*p* = 0.039), while emotion recognition showed visual but not statistical evidence of bias (*p* = 0.21). After trim-and-fill adjustment, effect sizes decreased modestly from *d* = 0.53 to 0.34 for emotion recognition and from *d* = 0.54 to 0.43 for theory of mind, yet remained significant. Bias assessment for executive function (k = 7) was inconclusive due to limited power. Overall, findings suggest the robustness of SPS intervention effects, though some inflation due to selective reporting cannot be ruled out. Future studies should strengthen transparency through preregistration and open data practices.

## 4. Discussion

This systematic review and meta-analysis provide the first quantitative synthesis of evidence on SPS interventions for children with ASD. Across 19 studies, SPS interventions produced moderate and statistically significant improvements in SPS competence, confirming their potential to enhance core social-emotional abilities. Subgroup analyses further showed that interventions led by teachers or collaborative teams in school settings yielded the strongest effects, highlighting the critical role of ecological validity. Beyond SPS competence, these interventions also generated moderate-to-large gains across related SEL domains, including social skills, emotion recognition, theory of mind, and executive function, demonstrating their broad and cross-domain impact. Collectively, these findings highlight SPS as a promising, integrative framework for promoting social-emotional functioning in ASD. Nevertheless, variations in methodological quality, with only a subset of studies meeting high evidence standards, limit the strength and generalizability of these conclusions.

### 4.1. Summary of Key Findings

Our review shows that SPS interventions represent an emerging and expanding focus within ASD education. Most studies were published between 2010 and 2019, reflecting growing scholarly and practical interest in the association between social-emotional competence and quality of life (Cohen, 2006; Osher et al., 2016). This trend highlights the need to systematically integrate SPS and SEL frameworks into educational curricula for children with ASD. However, all identified studies were conducted in high-income countries or regions, revealing a clear geographical and demographic imbalance. The absence of research in low and middle-income settings limits the cross-cultural generalizability of current findings (Durkin et al., 2015; Franz et al., 2017). Participant samples were also predominantly male (≈88%), leaving the effects of SPS interventions for females with ASD largely underexplored a critical gap for future research.

Implementation characteristics further varied across studies. SPS was most often delivered as a component within a broader SEL curriculum, though several studies positioned it as the central focus or part of a multi-component behavioral program. Most interventions occurred in school-based small group formats led by teachers or interdisciplinary teams. However, limited reporting on implementation fidelity, maintenance effects, and social validity remains a consistent weakness across the literature (Reichow et al., 2008; Wong et al., 2015). Without these data, the durability and ecological relevance of intervention outcomes remain uncertain.

### 4.2. Interpretation of Findings

This meta-analysis provides the first quantitative evidence that SPS interventions produce significant and broad benefits for children and adolescents with ASD. Across five outcome domains, SPS interventions yielded consistent, medium-sized effects, confirming their efficacy in enhancing core social emotional functioning. These results support the theoretical premise that SPS represents a pivotal skill driving cascading improvements across related domains, including social cognition, emotion understanding, and self-regulation. Interestingly, the consistency of intervention effects varied by domain: improvements in core SPS ability and executive function were robust and uniform, whereas gains in social skills, emotion recognition, and theory of mind showed significant heterogeneity. This suggests that while SPS interventions reliably strengthen foundational cognitive skills, the translation of these gains into broader social-emotional competencies may be influenced by contextual factors and individual differences (Pellicano, 2010).

Importantly, moderator analyses revealed that teacher-led, school-based interventions produced substantially larger effects compared to researcher-led or clinic-based programs. This finding underscores the critical role of ecological validity. This pattern suggests that social learning processes are best supported in environments offering repeated natural practice, authentic feedback, and peer interaction. In classrooms, teachers can incorporate SPS principles into daily routines, such as conflict resolution, cooperative learning, or classroom management, transforming interventions into sustained social teaching practices rather than discrete lessons (Kasari & Patterson, 2012). Conversely, researcher-led programs or clinic-based programs often lack these real-world contingencies, which likely accounts for their weaker transfer effects (Rotheram-Fuller et al., 2010).

The cross-domain gains observed in emotion recognition, theory of mind, and executive function further illuminate the mechanisms underlying SPS interventions. These outcomes imply that SPS training engages both cognitive and affective systems, helping children integrate reasoning, emotion, and behavior into a unified decision-making process (Fisher & Happé, 2005). Importantly, recent micro-level research offers mechanistic support for these macro-level findings. McNair et al. (2024) demonstrated that SPS is not a monolithic skill but a reasoning process that integrates multiple social-cognitive abilities. Together, these results suggest that SPS interventions promote cognitive restructuring of social reasoning, producing generalized and durable benefits beyond task-specific skill acquisition.

Finally, the results of publication bias analyses suggest that the effects on emotion recognition and theory of mind may be modestly overestimated. However, adjusted estimates continue to demonstrate statistical significance, affirming the overall robustness of the intervention effects while underscoring the importance of future replication studies to precisely determine their magnitude (Egger et al., 1997; Sterne & Egger, 2001).

### 4.3. Implications and Contributions

The findings from this meta-analysis support the positioning of SPS interventions as a valuable evidence-based practice for children with ASD. Based on our moderator analysis, which showed that teacher-led interventions in school settings, practitioners should use approaches that fit naturally into everyday settings (Rotheram-Fuller et al., 2010). Incorporating SPS instruction in the classroom enables modeling, peer practice, and instant feedback, which are key to helping skills transfer beyond isolated training settings (Kasari & Patterson, 2012). Therefore, practitioners should regularly include elements like parent training and community activities, and evaluating the effectiveness based on their impact in daily life (Dale et al., 2022; McConachie et al., 2018).

Moreover, SPS can function as a modular component within comprehensive SEL or cognitive-behavioral programs, enhancing its scalability across educational settings (Durlak et al., 2011). Its consistent impact supports its consideration for inclusion within Individualized Education Programs (IEPs) and broader school-based frameworks, such as Positive Behavioral Interventions and Supports (PBIS) or CASEL-aligned curricula. Educators can adapt the cognitive steps of problem solving, identifying problems, generating alternatives, and evaluating consequences into explicit classroom strategies aligned with everyday learning goals. Recent evidence shows that such school-based SPS interventions can reduce teacher-reported problem behaviors by up to 50% while increasing academic engagement (Peng et al., 2024). Consistent improvements in executive function suggest that integrating cognitive support (e.g., planning, inhibition, flexibility) within SPS training may produce synergistic benefits (Pellicano, 2012). Embedding these cognitive scaffolds within meaningful social contexts not only enhances transfer but also reflects a behavioral cognitive synthesis increasingly recognized as essential for effective ASD intervention. Taken together, these findings justify the inclusion of SPS training in teacher preparation, professional development, and clinical practice guidelines.

Theoretically, this meta-analysis advances a social-cognitive integration framework for understanding intervention effects in ASD. Unlike generic social skills training, SPS interventions are explicitly grounded in the social information processing model (Crick & Dodge, 1994), which conceptualizes social behavior as the product of sequential cognitive steps involving perception, interpretation, response generation, and evaluation. By quantifying effects across multiple domains, this review offers the first large-scale empirical validation of SPS as a central mediating process linking cognitive and emotional development in ASD (Chan & Leung, 2022).

The findings further support a collaborative social-cognitive model, emphasizing that social challenges in ASD stem from interdependent deficits in executive function, perspective taking, and metacognitive problem-solving rather than isolated behavioral impairments. SPS interventions appear effective precisely because they target this underlying cognitive network, promoting internalized reasoning and emotional regulation instead of surface-level behavioral compliance (Pellicano, 2010). By reframing intervention from training behaviors to cultivating reasoning processes, this meta-analysis deepens theoretical understanding and provides a mechanistic foundation for designing ecologically valid, cognitively informed SEL interventions (Lerner et al., 2012).

### 4.4. Limitations and Future Directions

Several limitations should be consideration.

First, generalizability is limited by sampling bias and uneven methodological rigor. Most studies were conducted in high-income countries, and approximately two-thirds did not fully meet WWC standards due to inadequate randomization, small samples, or incomplete reporting. Furthermore, the heavy reliance on subjective informant ratings (e.g., teacher or parent reports) for outcomes like social skills carries a risk of rater bias and may inflate effect sizes compared to blinded, performance-based assessments.

Second, publication bias for emotion recognition and theory of mind outcomes indicates a field-wide positive-result bias, potentially overestimating effects. Large-scale, preregistered trials with open data sharing and the inclusion of null findings are necessary to improve evidence transparency and reproducibility.

Third, the limited reporting of fidelity, maintenance, and social validity constrains understanding of sustainability. Without long-term follow-up data, it remains unclear whether SPS gains endure to become self-sustaining competencies as children develop.

Future research should prioritize three key areas: (1) Methodological rigor: adopting rigorous experimental designs with blinded, performance-based outcome measures; (2) Ecological validity: developing and testing teacher-led models in diverse cultural contexts; and (3) Longitudinal design: incorporating follow-up assessments to examine the durability and real-world transfer of intervention effects.

## 5. Conclusions

This systematic review and meta-analysis provide the first comprehensive quantitative synthesis of SPS interventions for children with ASD. The findings reveal consistent, moderate-to-strong improvements in SPS competence and related social–emotional domains, including social skills, emotion recognition, theory of mind, and executive function. These results support SPS as a core mechanism within the SEL framework that integrates cognition, emotion, and behavior. Crucially, teacher-led, school-based delivery emerged as most effective, emphasizing the paramount value of ecological validity. When SPS instruction is woven into classroom interactions, children benefit from authentic social practice, feedback, and peer modeling that promote lasting generalization. Despite methodological variability and limited cultural diversity, this meta-analysis establishes a solid empirical foundation for future inquiry. Advancing this field will require ecologically grounded, fidelity-monitored, and collaboratively implemented interventions that connect theory-driven design with real-world educational contexts. Collectively, the evidence identifies SPS as a promising, scalable, and mechanism-based approach for fostering meaningful social–emotional growth in children with ASD.

## Figures and Tables

**Table 1:** Summary of included studies and intervention characteristics.

Study	Participant Characteristics				Intervention Characteristics					Study Characteristics				Outcome Variables & Informants				
	Country	N	Male	Age	Setting	Program	Delivery	Duration	Role of SPS	Design	Tx Fid	Fol-Up	Soc Val	SPS	SS	ER	ToM	EF
Antshel et al. (2011)	USA	83	74	8–12	×	SAEI	SG by GS/team	1/week; 60 min/peer; 10w	Comp of SEL	Quasi	×	+	+	×	PR	×	×	×
Bauminger (2002)	Israel	15	11	8–17	School	ICPS	SG by Ts/team	2/week; 3 h/peer; 28w	Primary	Quasi	+	×	+	TR	TR, O	TR	×	×
Bauminger (2007a)	Israel	26	24	6–12	School	SST	SG by Ts/team	2/week; 90 min/peer; 28w	Comp of SEL	Quasi	×	×	+	TR	TR, O	TR, DA	TR, DA	TR, DA
Bauminger (2007b)	Israel	19	18	7–12	School	SST	I by Ts/team	2/week; 3 h/peer; 28w	Comp of SEL	Quasi	×	×	+	TR	×	TR, DA	×	×
Bauminger et al. (2013)	Israel	22	18	8–12	School	CBT- CI	SG by Ts	1/week; 45 min/peer; 12w	Multi	Quasi	×	×	+	TR, O	TR, O	×	TR, O	×
Beaumont and Sofronoff (2008)	Australia	49	44	7–11	University laboratory	JDTP	SG by GS/team	1/week; 60 min/peer; 9w	Multi	RCT	+	+	×	×	PR	TR, DA	×	×
Bonete et al. (2016)	Spain	37	33	7–13	Community rehabilitation center	SCI-Children	SG by Rs	1/week; 60 min/peer; 10w	Primary	Quasi	+	×	×	CR, DA	CR, DA	CR, DA	CR, DA	×
Chou (2020)	China	44	37	12–16	School	NOSE	I, SG by Rs/Ts	1/week; 15w	Primary	RCT	+	×	×	TR	×	×	×	×
Chou (2024)	China	76	56	12–16	School	BoB	SG by Ts	1/week; 2 h/peer; 12w	Multi	RCT	+	×	×	TR	TR	TR	×	×
Einfeld et al. (2018)	Australia	84	75	8–15	School	SAS	SG by Ts/team	1/week; 90 min/peer; 13w	Comp of SEL	Quasi	+	+	×	RR, DA	TR, PR	TR, PR	×	×
Eden and Oren (2021)	Israel	58	55	5–6	Preschool	CBT	SG by Rs	1/week; 30 min/peer; 8w	Primary	RCT	+	×	×	×	RR	RR, O	RR, O	×
Hochhauser et al. (2018)	Israel	61	55	12–18	School	CONTACT	SG by Rs	1/week; 60 min/peer; 6w	Primary	RCT	+	+	+	RR	RR	×	×	×
Kenworthy et al. (2014)	USA	67	59	7–11	School	UOT	SG by team	1/week; 30–40 min/peer; 28w	Multi	RCT	+	+	+	TR	TR, PR	×	×	TR, PR, DA
Koning et al. (2013)	Canada	15	15	10–12	×	CBT	SG by Rs	1/week; 120 min/peer; 15w	Comp of SEL	RCT	+	×	×	×	PR	PR	×	×
Lee et al. (2019)	USA	8	7	7–8	University laboratory	CBI	I/SG by GS	1/week; 45 min/peer; 14w	Comp of SEL	Quasi	×	+	+	×	PR	PR	×	×
Solomon et al. (2004)	USA	18	18	8–12	University center	SAEI	SG by team	1/week; 90 min/peer; 20w	Comp of SEL	RCT	×	×	+	CR, DA	×	CR, DA	CR, DA	×
Stichter et al. (2010)	USA	27	27	11–14	University laboratory	SCI	SG by Ts	2/week; 60 min/peer; 10w	Comp of SEL	Quasi	×	×	×	TR	PR	TR	TR, DA	PR
Stichter et al. (2012)	USA	20	19	6–10	University laboratory	SCI-E	SG by GS	2/week; 60 min/peer; 10w	Comp of SEL	Quasi	×	×	×	TR, DA	TR, PR	TR, DA	TR, DA	PR
Szumski et al. (2019)	Poland	12	8	4–7	Preschool	ICPS	SG by GS	5/week; 20 min/peer; 8w	Primary	RCT	+	×	×	TR	TR, DA	×	TR, DA	×

Note. + = Yes/Assessed/Reported; × = No/Not Assessed/Not Reported. BoB = Bug-out Bags; CBI = Cognitive Behavioral Intervention; CBT = Cognitive Behavioural Therapy; CBT-CI = CBT-Combined Intervention; Comp of SEL = SPS as a component of a broader SEL curriculum; CONTACT = Conflict Orientation and Negotiation Training in Children and Teens; CR = Clinician rating; DA = Direct assessment; Design = Study Design; Duration = Intervention duration and frequency; EF = Executive Function; ER = Emotion Recognition; Fol-Up = Follow-up assessment; GS = Graduate students; I = Individual delivery; ICPS = I Can Problem Solve; JDTP = The Junior Detective Training Program; Multi = SPS as part of multi-component program; NOSE = Navigation of Social Engagement Project; O = Observation; PR = Parent rating; Primary = SPS as the primary focus; Quasi = Quasi-experimental design; RCT = Randomized controlled trial; RR = Researcher rating; Rs = Researchers; SAS = The Secret Agent Society Program; SCI = Social Competence Intervention; SG = Small group delivery; Soc Val = Social validity; SPS = Social Problem-Solving; SST = Social Skills Training; Team = Multidisciplinary team; ToM = Theory of Mind; TR = Teacher rating; Ts = Teachers; Tx fid = Treatment fidelity; UOT = Unstuck and On Target.

**Table 2:** Moderator analyses of SPS intervention effects.

Moderator Variable	Subgroup	*k*	*ES*	95% *CI*		*I*^2^ (%)	*Q*	*df*	*p*
				Lower	Upper				
Intervention Setting	School	10	0.61	0.43	0.79	0	3.46	1	0.06
	Non-school	4	0.26	−0.05	0.58	0			
Implementer Type	Teacher	4	0.72	0.43	1.01	0.48	7.31 *	2	0.03
	Researcher	4	0.20	−0.08	0.49	0			
	Team	6	0.63	0.38	0.88	0			
Session frequency	1 times/week	8	0.61	0.41	0.81	9.72	1.63	1	0.20
	≥2 times/week	6	0.40	0.14	0.65	0			
Session Length	20–60 min	7	0.41	0.20	0.62	10.68	3.33	2	0.18
	60–90 min	3	0.58	0.22	0.94	0			
	>90 min	4	0.77	0.44	1.10	0			
Total Duration	<8 weeks	2	0.24	−0.14	0.63	0	3.04	2	0.21
	8–12 weeks	6	0.52	0.29	0.75	21.53			
	≥12 weeks	6	0.66	0.40	0.91	0			
Role of SPS Component	Primary	5	0.44	0.17	0.70	33.06	2.40	2	0.30
	Comp of SEL	6	0.47	0.22	0.72	0			
	Multi	3	0.74	0.43	1.05	0			

Notes. *k* = The number of *ES*; *ES* = Cohen’s *d*; *CI* = confidence interval; *df* = degree of freedom; *Q* = *Q* test heterogeneity; * = statistically significant.

## Data Availability

The data that support the findings of this study are available from the corresponding authors upon reasonable request. Supplementary datasets, coding protocols, and analysis scripts are provided in the Supplementary Materials.

## Supplementary Materials

The following supporting information can be downloaded at: https://www.mdpi.com/article/10.3390/bs15121708/s1, Table S1: Full Search Strings for Each Database; Table S2: The description of codes; Table S3: Methodological Quality Assessment of Included Studies Based on WWC Standards; Table S4: Summary of Meta-Analytic Effects for SPS Competence and SEL-Related Outcomes; Table S5: Publication bias tests and trim-and-fill results. Table S6: PRISMA_Checklist.
