# Supplementary material for: Effectiveness of Social Problem-Solving Interventions for Children with Autism Spectrum Disorder: A Systematic Review and Meta-Analysis
Source: Behav Sci (Basel). 2025 Dec 10;15(12):1708. doi: 10.3390/bs15121708 (PMC12729265; doi:10.3390/bs15121708)
Supplement: Supplementary file 1 [file behavsci-15-01708-s001.zip › Table S1. Full Search Strings for Each Database.pdf]

Table S1. Full Search Strings for Each Database

Database	Search String	Records (n)
ERIC (ProQuest)	(SU Autism Spectrum Disorder OR SU autistic disorder OR SU Autism) AND (SU children OR SU young children OR SU child) AND (SU social problem solving OR SU problem solving OR SU cognitive-behavior) AND (SU intervention OR SU treatment OR SU therapy OR SU behavior intervention)	17
APA PsycINFO	(SU Autism Spectrum Disorder OR SU autistic disorder OR SU Autism) AND (SU children OR SU young children OR SU child) AND (SU social problem solving OR SU problem solving OR SU cognitive-behavior) AND (SU intervention OR SU treatment OR SU therapy OR SU behavior intervention)	172
Academic Search Premier	(SU Autism Spectrum Disorder OR SU autistic disorder OR SU Autism) AND (SU children OR SU young children OR SU child) AND (SU social problem solving OR SU problem solving OR SU cognitive-behavior) AND (SU intervention OR SU treatment OR SU therapy OR SU behavior intervention)	12
Psychological and Behavioral Sciences Collection	(SU Autism Spectrum Disorder OR SU autistic disorder OR SU Autism) AND (SU children OR SU young children OR SU child) AND (SU social problem solving OR SU problem solving OR SU cognitive-behavior) AND (SU intervention OR SU treatment OR SU therapy OR SU behavior intervention)	4
Social Science Citation Index	TS= ("social problem solving" OR "problem solving" OR "cognitive behavior") AND TS= ("autism spectrum disorder" OR "Autistic Disorder" OR "Autism") AND TS= ("children" OR "young children" OR "child") AND TS= ("Intervention" OR "behavior intervention" OR "treatment" OR "therapy")	1,141
APA PsycArticles (PsycNET)	SU (Autism Spectrum Disorder OR autistic disorder OR Autism) AND SU (children OR young children OR child) AND SU (social problem solving OR problem solving OR cognitive-behavior) AND SU (intervention OR treatment OR therapy OR behavior intervention)	4
PubMed	(((Autism Spectrum Disorder[Title/Abstract]) OR (autistic disorder[Title/Abstract]) OR (Autism[Title/Abstract])) AND ((children[Title/Abstract]) OR (young children[Title/Abstract]) OR (child[Title/Abstract]))) AND ((social problem solving[Title/Abstract]) OR (problem solving[Title/Abstract]) OR (cognitive-behavior[Title/Abstract])) AND ((intervention[Title/Abstract]) OR (treatment[Title/Abstract]) OR (therapy[Title/Abstract]))	87
Total		1437

Note. Search strings were adapted to the specific syntax of each database. For example, TS= denotes a Topic Search in Web of Science; [Title/Abstract] denotes a search in the title or abstract fields in PubMed; DE or SU denotes a search using Subject Terms or Descriptors in ProQuest and EBSCOhost databases.
