# Supplementary material for: Effectiveness of Social Problem-Solving Interventions for Children with Autism Spectrum Disorder: A Systematic Review and Meta-Analysis
Source: Behav Sci (Basel). 2025 Dec 10;15(12):1708. doi: 10.3390/bs15121708 (PMC12729265; doi:10.3390/bs15121708)
Supplement: Supplementary file 1 [file behavsci-15-01708-s001.zip › Table S2. The description of codes.pdf]

Table S2. The description of codes

Category	Code Name	Coding Rule	Example or Coding Criteria
Participant Characteristics	Country	Country or region where the study was conducted.	"United States," "Israel," "China."
	N (Sample Size)	Total number of participants with ASD included in the analysis.	Record total N per intervention arm.
	Male	Percentage or number of male participants in the sample.	Extract exact count or calculate from total.
	Age Range	Reported chronological age of participants.	"6–12 years" or "Mean = 9.3 years."
Intervention Characteristics	Setting	Primary intervention context.	1 = School; 2 = University lab; 3 = Community center; 4 = Preschool.
	Program Name	The specific program or curriculum name.	e.g., ICPS, SCI, SAS, UOT, CBT, SST.
	Delivery Format	Mode and implementer of delivery.	"SG by Ts/team," "I by Rs," "SG by GS."
	Frequency & Duration	Frequency of sessions per week, session length, and total program duration.	"1/week; 60 min; 10 weeks."
	Role of SPS	The functional position of the SPS component in the intervention.	1 = Primary focus; 2 = Component of SEL; 3 = Part of multi-component program.
Study Design	Design Type	Experimental design used.	1 = RCT; 2 = Quasi-experimental.
	Treatment Fidelity (Tx fid)	Whether the study reported monitoring of intervention fidelity.	"+" = Reported; "×" = Not reported.
	Follow-Up (Fol-Up)	Whether follow-up data were collected after intervention.	"+" = Follow-up conducted; "×" = No follow-up.
	Social Validity (Soc Val)	Whether social validity or acceptability outcomes were assessed.	"+" = Assessed; "×" = Not assessed.
Outcome Domains and Informants	SPS Competence (SPS)	Direct or indirect measures of social problem-solving ability.	"I Can Problem Solve Questionnaire," "SPS Questionnaire."
	Social Skills (SS)	Behavioral or rating-based assessments of social functioning.	"Social Skills Rating System (SSRS)."
	Emotion Recognition (ER)	Measures assessing recognition or labeling of emotional expressions.	"DANVA," "Emotion Recognition Test."
	Theory of Mind (ToM)	Performance on false-belief or perspective-taking tasks.	"Reading the Mind in the Eyes Test."
	Executive Function (EF)	Measures of planning, working memory, or cognitive flexibility.	"BRIEF," "Tower of London."
	Informant Type	Source of outcome data.	TR = Teacher rating; PR = Parent rating; RR = Researcher rating; CR = Clinician rating; DA = Direct assessment; O = Observation.

Note. All variables were coded based on explicit information reported in the original studies.
(+) = Yes / Reported / Assessed; (×) = No / Not Reported. When data were not explicitly stated but could be inferred from study descriptions (e.g., session frequency), coders reached consensus through discussion. Ambiguous cases were coded as missing.
