# Supplementary material for: Effectiveness of Social Problem-Solving Interventions for Children with Autism Spectrum Disorder: A Systematic Review and Meta-Analysis
Source: Behav Sci (Basel). 2025 Dec 10;15(12):1708. doi: 10.3390/bs15121708 (PMC12729265; doi:10.3390/bs15121708)
Supplement: Supplementary file 1 [file behavsci-15-01708-s001.zip › Table S3. Methodological Quality Assessment of Included Studies Based on WWC Standards.pdf]

Table S3. Methodological Quality Assessment of Included Studies Based on WWC Standards

Study	Assignment of participants	Sample attrition	Baseline equivalence	Outcome requirements				Confounding factors	Overall ratings
				Face validity	Reliability	Over alignment	Outcome collection		
Antshel et al. (2011)	N	R	Y	Y	Y	Y	Y	Y	R
Bauminger (2002)	N	Y	NA	Y	Y	Y	Y	N	N
Bauminger (2007a)	N	R	Y	Y	Y	Y	Y	Y	R
Bauminger (2007b)	N	R	NA	Y	Y	Y	Y	N	N
Bauminger et al. (2013)	N	R	N	Y	Y	Y	Y	Y	N
Beaumont & Sofronoff (2008)	Y	Y	N	Y	Y	Y	Y	Y	R
Bonete et al. (2016)	N	Y	NA	Y	Y	Y	Y	N	N
Chou (2020)	Y	Y	N	Y	Y	Y	Y	Y	R
Chou (2024)	Y	Y	R	Y	Y	Y	Y	Y	Y
Eden & Oren (2021)	Y	Y	Y	Y	Y	Y	Y	Y	Y
Einfeld et al. (2018)	N	R	Y	Y	Y	Y	Y	Y	R
Hochhauser et al. (2018)	Y	Y	Y	Y	Y	Y	Y	Y	Y
Kenworthy et al. (2014)	Y	Y	Y	Y	Y	Y	Y	Y	Y
Koning et al. (2013)	Y	R	N	Y	Y	Y	Y	Y	R
Lee et al. (2019)	N	Y	NA	Y	Y	Y	Y	Y	N
Solomon et al. (2004)	Y	Y	Y	Y	Y	Y	Y	Y	Y
Stichter et al. (2010)	N	Y	NA	Y	Y	Y	Y	N	N
Stichter et al. (2012)	N	Y	NA	Y	Y	Y	Y	N	N
Szumski et al. (2019)	Y	Y	Y	Y	Y	Y	Y	Y	Y

Note. Y = meets the standard without reservations; N = does not meet the standard; R = meets the standard with reservations, NA = not applicable.
