# Supplementary material for: Effectiveness of Social Problem-Solving Interventions for Children with Autism Spectrum Disorder: A Systematic Review and Meta-Analysis
Source: Behav Sci (Basel). 2025 Dec 10;15(12):1708. doi: 10.3390/bs15121708 (PMC12729265; doi:10.3390/bs15121708)
Supplement: Supplementary file 1 [file behavsci-15-01708-s001.zip › Table S4. Summary of Meta-Analytic Effects for SPS Competence and SEL-Related Outcomes.pdf]

Table S4. Summary of Meta-Analytic Effects for SPS Competence and SEL-Related Outcomes

Domain	k	Model	Effect Size (d)	95% CI	SE	p	I^2 (%)	Q (p)	Interpretation
Social Problem Solving	14	Fixed	0.53	[0.15, 1.01]	0.08	< .05	0.0	12.02 (.52)	Moderate, significant
Emotion Recognition	16	Random	0.53	[0.32, 0.74]	0.10	< .05	37.5	24.00 (.06)	Moderate, significant
Executive Function	7	Fixed	0.56	[0.35, 0.89]	–	< .001	0.0	3.9 (.54)	Medium, robust
Social Skills	20	Random	0.65	[0.24, 1.42]	0.10	< .05	43.6	33.6 (.02)	Moderate-to-large
Theory of Mind	10	Random	0.54	[0.08, 1.71]	0.17	< .01	54.6	19.81 (.01)	Moderate, significant

Note. d = Cohen's d; k = number of effect sizes; CI = confidence interval; SE = standard error. Analyses were conducted using fixed- or random-effects models depending on heterogeneity (I^2, Q, and p values).
