# Supplementary material for: Effectiveness of Social Problem-Solving Interventions for Children with Autism Spectrum Disorder: A Systematic Review and Meta-Analysis
Source: Behav Sci (Basel). 2025 Dec 10;15(12):1708. doi: 10.3390/bs15121708 (PMC12729265; doi:10.3390/bs15121708)
Supplement: Supplementary file 1 [file behavsci-15-01708-s001.zip › Table S5. Publication bias tests and trim-and-fill results.pdf]

Table S5. Publication bias tests and trim-and-fill results

Outcome	k	Egger's Test (p)	Trim-and-Fill Adjusted g	95% CI	Fail-Safe N	Interpretation
Social Problem Solving	14	0.74	0.53	[0.32, 0.74]	141 (>80)	No publication bias
Social Skills	20	0.064	0.62	[0.38, 0.85]	387 (>110)	Marginal asymmetry, effect robust
Emotion Recognition	16	0.21	0.34	[0.10, 0.59]	95 (>60)	Possible bias, adjusted effect significant
Theory of Mind	10	0.039	0.43	[0.10, 0.81]	48 (<60)	Significant bias, cautious interpretation
Executive Function	7	0.74	0.56	[0.20, 0.92]	37 (<45)	Inconclusive (low power)

Note. Egger's test assesses funnel plot asymmetry; lower p-values indicate potential bias. Trim-and-fill adjusted g reflects recalculated effect sizes after imputing missing studies. Fail-safe N indicates the number of null studies required to reduce the result to non-significance. Interpretations are based on combined visual, statistical, and sensitivity analyses.
