# Supplementary material for: Effectiveness of Social Problem-Solving Interventions for Children with Autism Spectrum Disorder: A Systematic Review and Meta-Analysis
Source: Behav Sci (Basel). 2025 Dec 10;15(12):1708. doi: 10.3390/bs15121708 (PMC12729265; doi:10.3390/bs15121708)
Supplement: Supplementary file 1 [file behavsci-15-01708-s001.zip › Table_S6_PRISMA_Checklist.pdf]

PRISMA Checklist

Title: Effectiveness of Social Problem-Solving Interventions for Children with Autism Spectrum Disorder: A Systematic Review and Meta-Analysis

Section and Topic	Item #	Checklist item	Location in Manuscript
TITLE	1	Identify the report as a systematic review.	Title page
ABSTRACT	2	See the PRISMA 2020 for Abstracts checklist.	Abstract
INTRODUCTION - Rationale	3	Describe the rationale for the review in the context of existing knowledge.	Section 1.1 Background and Significance
INTRODUCTION - Objectives	4	Provide an explicit statement of the objective(s) or question(s) the review addresses.	Section 1.3 Research Questions and Aims
METHODS - Eligibility criteria	5	Specify inclusion and exclusion criteria for the review and how studies were grouped for the syntheses.	Section 2.2 Inclusion and Exclusion Criteria
METHODS - Information sources	6	Specify all databases, registers, websites, organisations, reference lists and other sources searched, and date last searched.	Section 2.1 Search Strategy; Table S1
METHODS - Search strategy	7	Present the full search strategies for all databases, including any filters and limits used.	Supplementary Materials, Table S1
METHODS - Selection process	8	Specify the methods used to decide whether a study met the inclusion criteria, including reviewer process and independence.	Section 3.1 Study Selection; Figure 1
METHODS - Data collection process	9	Specify the methods used to collect data from reports, including number of reviewers and consensus procedures.	Section 2.3 Data Extraction and Coding; Table S2
METHODS - Data items (10a)	10a	List and define all outcomes for which data were sought and specify selection methods.	Section 2.5 Data Synthesis and Statistical Analysis
METHODS - Data items (10b)	10b	List and define all other variables for which data were sought (e.g., participant characteristics, intervention details).	Section 2.3 Data Extraction and Coding; Table 1
METHODS - Study risk of bias assessment	11	Specify the methods used to assess risk of bias in included studies.	Section 2.4 Study Quality and Risk of Bias Assessment
METHODS - Effect measures	12	Specify for each outcome the effect measures used (e.g., SMD, mean difference).	Section 2.5 Data Synthesis and Statistical Analysis
METHODS - Synthesis methods (13a–f)	13a–f	Describe synthesis processes, data preparation, tabulation, model type, heterogeneity assessment, and sensitivity analyses.	Section 2.5 Data Synthesis and Statistical Analysis; Section 3.5–3.6
METHODS - Reporting bias assessment	14	Describe methods used to assess risk of bias due to missing results.	Section 3.6 Publication Bias and Sensitivity Analyses
METHODS - Certainty assessment	15	Describe any methods used to assess certainty (confidence) in the body of evidence.	Not assessed
RESULTS - Study selection (16a)	16a	Describe the search and selection process, ideally using a flow diagram.	Section 3.1 Study Selection; Figure 1
RESULTS - Study selection (16b)	16b	Cite studies that met inclusion criteria but were excluded and explain why.	Section 3.1 Study Selection
RESULTS - Study characteristics	17	Cite each included study and present its characteristics.	Section 3.2 Characteristics of Included Studies; Table 1

RESULTS - Risk of bias in studies	18	Present assessments of risk of bias for each included study.	Section 3.3 Methodological Quality; Table S3
RESULTS - Results of individual studies	19	Present summary statistics for each group and effect estimates.	Supplementary Table S4; Figures 2–3
RESULTS - Results of syntheses (20a–d)	20a–d	Summarize synthesis results, including heterogeneity, subgroup, and sensitivity analyses.	Section 3.4–3.6; Figures 2–4; Table 2; Tables S3–S4
RESULTS - Reporting biases	21	Present assessments of risk of bias due to missing results for each synthesis.	Section 3.6 Publication Bias and Sensitivity Analyses; Table S5
RESULTS - Certainty of evidence	22	Present assessments of certainty (confidence) in the body of evidence for each outcome.	Not assessed
DISCUSSION (23a–d)	23a–d	Interpret results, discuss limitations of evidence and review process, and implications for practice and research.	Section 4.1–4.4 Discussion
OTHER INFORMATION - Registration and protocol	24a–c	Provide registration information and describe any protocol amendments.	Section 2.1 Search Strategy; PROSPERO registration
OTHER INFORMATION - Support	25	Describe sources of financial or non-financial support for the review.	Funding statement
OTHER INFORMATION - Competing interests	26	Declare any competing interests of authors.	Conflict of Interest statement
OTHER INFORMATION - Availability of data and materials	27	Report availability of data, code, and materials.	Data Availability statement; Supplementary Materials
